# Development of a fluorescent reporter strain to facilitate studies of *Borrelia burgdorferi* pathogenesis

**DOI:** 10.1128/spectrum.02290-25

**Published:** 2026-06-01

**Authors:** Annalisa B. Huckaby, Maria de la Paz Gutierrez, Ching Wooen Sze, Barrett-Anne Briggs, Kelly L. Weaver, Evita Yang, Carleena M. Rocuskie-Marker, Spencer R. Dublin, Joshua M. Hall, Samuel R. Ulicny, Gage M. Pyles, William T. Witt, Matthew S. Hudson, Craig A. Land, F. Heath Damron, Christopher L. Pritchett, Chunhao Li, Timothy Driscoll, Emel Sen-Kilic, Mariette Barbier

**Affiliations:** 1 Department of Microbiology, Immunology, and Cell Biology, School of Medicine, West Virginia University 5631, Morgantown, West Virginia, USA; 2 Vaccine Development Center, West Virginia University, Health Sciences Center 5631, Morgantown, West Virginia, USA; 3 Department of Oral Craniofacial Molecular Biology, Virginia Commonwealth University 6889, Richmond, Virginia, USA; 4 Department of Biology, West Virginia University 5631, Morgantown, West Virginia, USA; 5 College of Public Health, East Tennessee State University 4154, Johnson City, Tennessee, USA; Institute of Parasitology, Biology Centre, ASCR, Ceske Budejovice, Czechia; Rocky Mountain Laboratories, NIAID, NIH, Hamilton, Montana, USA

**Keywords:** *Borrelia burgdorferi*, GFP labeling, murine model, flow cytometry, detection models, Lyme disease

## Abstract

Lyme disease is a tick-borne disease caused by spirochetes of the *Borrelia burgdorferi sensu lato* complex. Murine models of *B. burgdorferi* infection allows for the assessment of bacterial dissemination and presence through culture of infected tissue and dark-field microscopic examination after a period of outgrowth. Although valuable, dark-field microscopy can be subjective, complex, and time-consuming. We propose that the limitations of current culture-screening methodologies could be overcome using a fluorescent *B. burgdorferi* stain for murine challenge studies. To test this, we generated A3-68*Δbbe02* pBBE22G-flgBpGFP, a strain of *B. burgdorferi* that expresses the fluorescent protein GFP encoded on the plasmid pBBE22G. We then validated that the A3-68*Δbbe02* pBBE22G-flgBpGFP strain (*Bb-*GFP) expressed detectable GFP *in vitro* and that it grew and had a similar infection profile in mice compared to its parental strain, A3-68*Δbbe02*. Next, we compared the accuracy and sensitivity of three different methods to detect *B. burgdorferi* from infected tissues: flow cytometry, dark-field microscopy, and PCR. In a *Bb-*GFP murine challenge model, screening of tissue cultures with flow cytometry exhibited sample positivity similar to that of traditional methods, with increased objectivity, speed, and efficiency. Overall, the *Bb*-GFP fluorescent strain and associated methodology described here provide additional resources for the study of *B. burgdorferi* pathogenesis.

**IMPORTANCE:**

Studying *Borrelia burgdorferi* pathogenesis can be challenging due to reliance on labor-intensive techniques such as dark-field microscopy to determine the infection status of animals. In this study, we demonstrated that flow cytometry could be used as an alternative approach, allowing for fast, reliable, and comparable results to those of dark-field microscopy in the context of mouse infection models.

## INTRODUCTION

*Borrelia burgdorferi,* a member of the Spirochaetaceae family, is one of the causative agents of the tick-borne disease known as Lyme disease (1). *B. burgdorferi* is classified as an enzootic pathogen infecting ticks of the *Ixodes* species and primary reservoirs such as small mammals (2). Humans and other mammals become incidental hosts infected with this pathogen through the bite of an infected tick (3). Lyme disease incidence rates have been steadily increasing (4), and it is currently the most common vector-borne disease in the United States (5). Without early detection and antibiotic intervention, the disease can progress into a variety of clinical manifestations, resulting in lifelong symptoms. Early detection is at times difficult due to heterogeneous presentation of symptoms and limited diagnostic accuracy during the early phases of the disease (6, 7). Furthermore, between 10% and 20% of patients who were diagnosed early and treated developed post-treatment Lyme disease (PTLD) symptoms (5). Taking these complications together with the absence of approved human Lyme vaccines, the need for the development of novel therapeutics for the prevention and treatment of Lyme disease is only increasing.

To accomplish the overarching goal of improving outcomes for individuals infected with *B. burgdoferi*, we need to better understand the pathogen during infection. Since its discovery in the 1980s, researchers have investigated the pathogenesis of this bacterium and established murine models of infection to study Lyme disease. A primary readout from infection is the detection of bacterial presence and dissemination in tissues. The standard for bacterial burden quantification is to plate tissue homogenates from infected animals on solid agar to determine the number of viable cells through colony-forming unit (CFU) enumeration. However, CFU enumeration of *B. burgdorferi* in preclinical models is not widely used due to several factors. Plating *B. burgdorferi* into semisolid BSK plates is time-consuming, and *B. burgdorferi* colonies are difficult to distinguish from eukaryotic debris as they are suspended within the agar and lack defined edges. Furthermore, *B. burgdorferi* is a slow-growing pathogen that requires a nutrient-rich medium, increasing the risk of contamination during the incubation period. Altogether, quantifying live *B. burgdorferi* in tissue during animal experiments involving large numbers of samples is challenging and is often replaced by other techniques.

Instead of live cell enumeration, *B. burgdorferi* presence is routinely assessed through the isolation of tissue, followed by qPCR amplification of *Borrelia*-specific genes and/or inoculation into *Borrelia*-specific culturing medium, such as BSK-II (3, 8–13). Quantitative PCR allows for the calculation of the number of bacteria as gene copy numbers in different organs, but it does not inform the viability of the bacteria. To address whether the spirochetes are alive, this technique can be complemented with tissue culture inoculation and screening via dark-field microscopy.

While assessment of culture positivity by dark-field microscopy is useful, it has a few shortcomings. First, these cultures are complex samples, making it difficult to distinguish *Borrelia* from eukaryotic cellular debris. Second, utilization of this qualitative method for determining positivity introduces person-to-person variability. Third, screening cultures via microscope is time-consuming and varies in length depending on the sample and experience of the examiner. We propose overcoming limitations of dark-field microscopy by using a GFP-labeled *B. burgdorferi* strain during murine challenge studies.

Fluorescent labeling of bacteria is a tool used throughout microbiology and immunology research (14). For *B. burgdorferi*, fluorescence has been previously used in various studies to investigate reporter genes, perform microscopy on infected samples both *in vitro* and *in vivo*, and conduct various flow-cytometry-based assays (15–22). We hypothesized that an additional use of a fluorescent *B. burgdorferi* strain could be to streamline organ culture screening efforts in the context of *B. burgdorferi* murine challenge studies by using flow cytometry as a method to assess sample positivity rather than a combination of dark-field microscopy and PCR.

In this work, we generated a GFP plasmid (pBBE22G-flgBpGFP) and confirmed that the addition of this plasmid to the A3-68Δ*bbe02* B31 *B. burgdorferi* strain did not alter the *in vitro* morphology, growth, or infectivity of *Bb*-GFP compared to the parental strain. Next, we demonstrated that *Bb-*GFP is capable of infecting ticks and that infected ticks retain GFP-positive *B. burgdorferi* through larval and nymphal stages. Lastly, we validated the use of GFP property as a method for flow cytometry-based culture screening and demonstrated high sample concordance with traditional screening methods, requiring less effort and time for *B. burgdorferi* identification. Altogether, this work demonstrates that GFP labeling of *B. burgdorferi* is beneficial for the identification and screening of spirochetes to assess culture positivity in both *in vitro* and *in vivo* samples. This new method will be highly valuable in ongoing research for drug and vaccine development efforts against *B. burgdorferi*.

## RESULTS

### Inclusion of pBBE22G-*flgBpGFP* has minimal effect on the growth and appearance of B31-A3-68Δ*bbe02*

**Fig 1:**
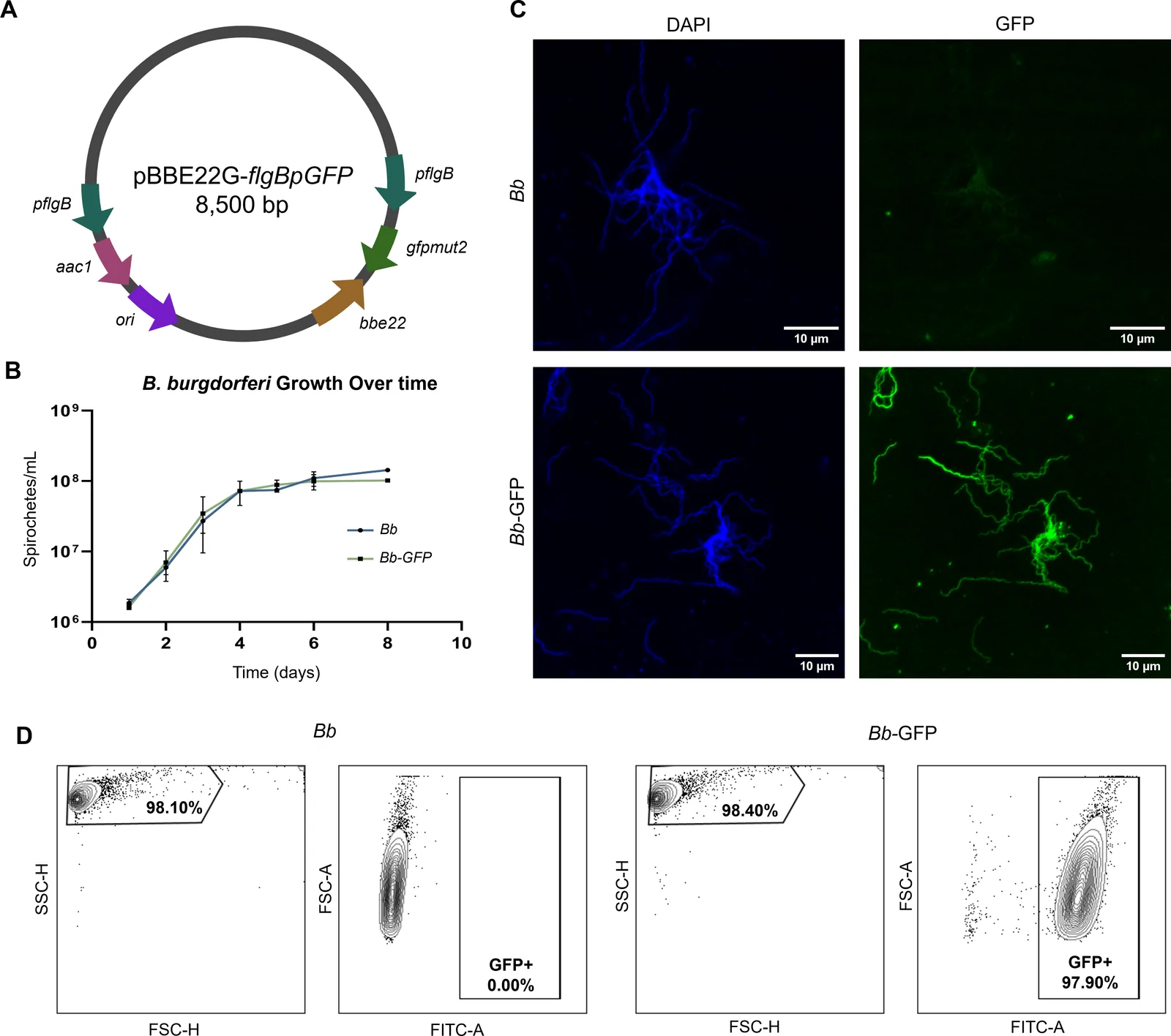
*In vitro* characterization of the *B. burgdorferi* GFP strain. (**A**) Annotated *B. burgdorferi* GFP plasmid pBBE22G-flgBpGFP, generated in BioRender. *bbe22*, encoding the nicotinamidase PncA; *gfpmut2*, encoding a green fluorescent protein (GFP) under the control of the *flgB* promoter (*pflgB*); *aac1*, encoding an N-acetyltransferase under the control of *pflgB*, conferring resistance to gentamicin. (**B**) *Growth of Bb* (A3-68Δ*bbe02*) and *Bb*-GFP (A3-68Δ*bbe02* pBBE22G-flgBpGFP) in BSK-II over time, enumerated via dark-field microscopy. Data are represented as mean ± SD. (**C**) DAPI-stained *B. burgdorferi* imaged via Nikon A1R/SIM fluorescence microscopy. (**D**) Representative images of unstained *B. burgdorferi* flow cytometry plots. Samples were gated based on forward and side scatter (SSC-H vs. FSC-H) and then on GFP positivity (FSC-A vs. FITC-A).

The fluorescent strain used in this study was generated in the A3-68Δ*bbe02* B31 background strain, herein referred to as *Bb*. First described by Rego et al., this B31 derivative strain harbors a deletion at the *bbe02* locus on plasmid lp25, allowing for an increased transformation rate while maintaining its virulence in mice (23). The pBBE22G-*flgBpGFP* shuttle vector was electroporated into A3-68Δ*bbe02* B31 to create A3-68Δ*bbe02* B31 pBBE22G-*flgBpGFP*, herein referred to as *Bb*-GFP. The pBBE22G-*flgBpGFP* shuttle vector encodes the GFP gene, a gentamicin resistance cassette, and the *bbe22* gene, all under the control of the commonly used *B. burgdorferi flgB* promoter (Fig. 1A). The gentamicin resistance cassette serves as an *in vitro* selection marker, while the *bbe22* gene encodes a murine virulence-required protein, PncA (24) (Fig. 1A).

Expression of recombinant proteins can often alter bacterial physiology and pathogenesis (14). To determine if the inclusion of the pBBE22G-*flgBpGFP* plasmid (GFP plasmid) affected *in vitro* growth, *Bb* and *Bb-*GFP strains were inoculated into Barbour-Stoenner-Kelly medium (BSK-II) with appropriate antibiotics, and spirochete concentration was monitored by dark-field microscopy over 8 days. No significant differences in growth were observed during this time (Fig. 1B). Next, to verify that the *Bb*-GFP strain expressed detectable levels of GFP, culture-grown *Bb* and *Bb*-GFP were imaged using confocal microscopy (Fig. 1C) and analyzed using flow cytometry (Fig. 1D). Both strains appeared to be similar in morphology (Fig. 1C), and expression of GFP in the *Bb*-GFP strain was detected by both microscopy (Fig. 1C) and flow cytometry (Fig. 1D).

One caveat of labeling bacteria using plasmid-encoded proteins is that bacterial plasmids can often be lost in the absence of selective pressure. To facilitate the study of *B. burgdorferi* during infection, it is important that the expression of GFP is stable over time and maintained in the absence of selective pressure. To test this, the *in vitro* stability of the pBBE22G-*flgBpGFP* plasmid (GFP plasmid) was assessed over time in the *Bb-*GFP strain in the presence and absence of the selective antibiotic gentamicin (Fig. S1A through D) using qPCR, flow cytometry, and microscopy. Using qPCR, we observed that the *gfp* copy number (cn) was maintained over 31 days in both conditions tested. As expected, cultures supplemented with the selective antibiotic had slightly higher *gfp* copy numbers over time; however, this difference was not statistically significant (Fig. S1A). GFP expression was monitored post-inoculation in both antibiotic conditions tested using flow cytometry (Fig. S1B) and microscopy (Fig. S1C and D). At day 31 post-inoculation, cultures inoculated with *Bb*-GFP in the presence of streptomycin or streptomycin + gentamicin had 92% and 94% GFP-positive *Bb* via flow cytometry, respectively (Fig. S1B). The flow cytometry data were corroborated by the microscopy analysis, in which GFP positivity was detected throughout the duration of the experiment (Fig. S1C and D). Together, these data indicate that both the GFP plasmid and GFP (*gfp*) expression in the *Bb*-GFP strain are maintained for at least 31 days *in vitro* in the presence or absence of selective antibiotics, making the strain stable for murine studies.

### Inclusion of the GFP plasmid does not alter infectivity in mice in a needle-*B. burgdorferi* infection model

**Fig 2:**
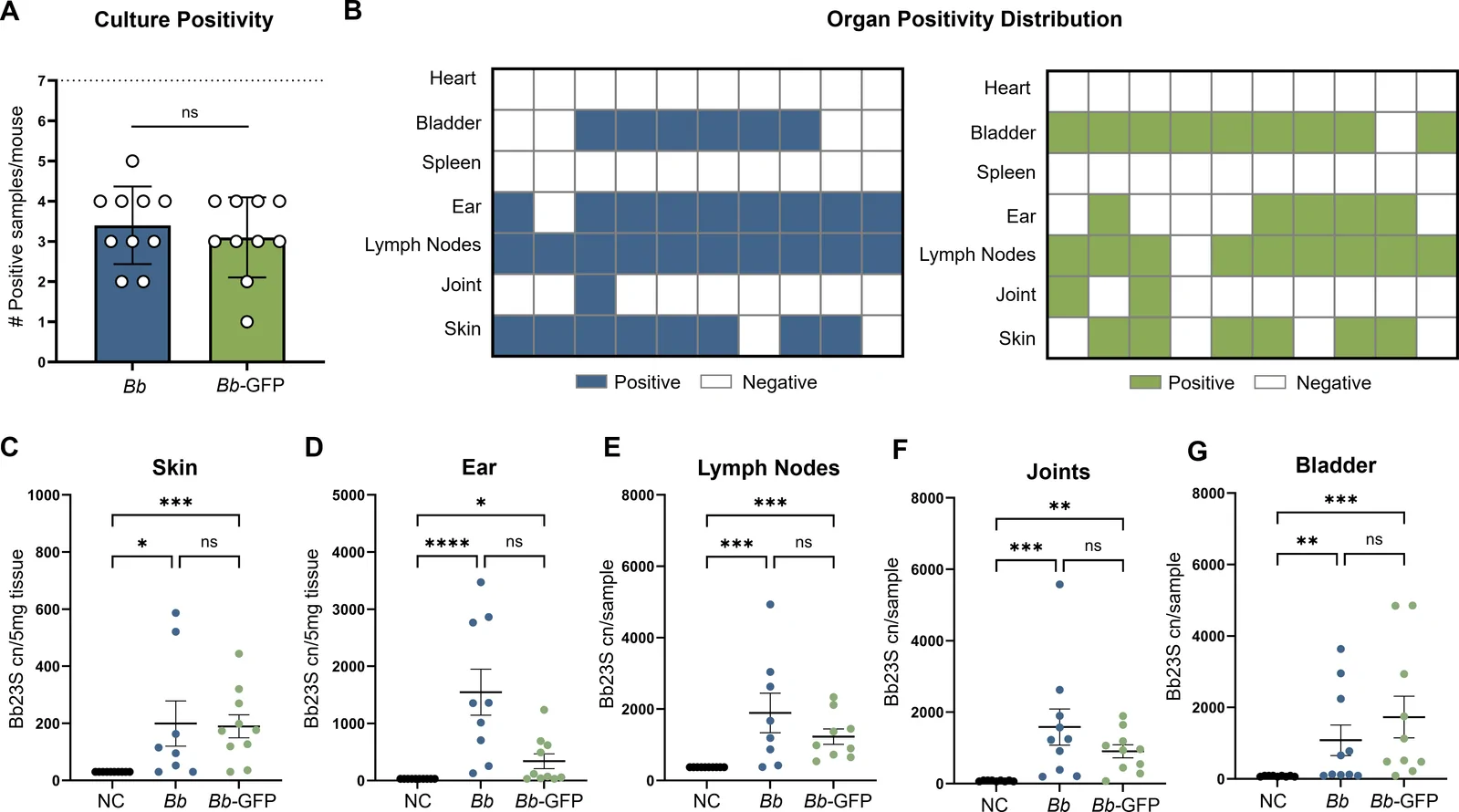
Murine challenge with *Bb* and *Bb*-GFP strains. (**A**) Number of culture-positive organs per mouse on day 14 post-challenge assessed via dark-field microscopy and PCR (*n* = 10). Data are represented as means ± SD. Dotted line at y = 7 signifies the maximum possible number of positive organs per mouse. Fisher’s exact test was performed. (**B**) Mouse organ distribution of culture positive/negative samples on day 14 after challenge. Each column represents samples collected from a single mouse, and each row represents an organ (heart, bladder, spleen, ear, lymph nodes, joint, and skin). 23S copies per sample determined via qPCR on skin (**C**), ear (**D**), lymph nodes (**E**), joints (**F**), and bladder (**G**) 14 days post-challenge (*n* = 8–10). NC denotes non-challenged mice. Outliers were removed following an outlier test. Data are represented as mean ± SEM. Kruskal-Wallis tests were performed for qPCR data. ns, not significant, *P < 0.05, **P < 0.01, ***P < 0.001, ****P < 0.0001.

To determine if inclusion of the GFP plasmid into the *Bb* strain affected murine virulence, 10-week-old C3H/HeN mice were challenged at the base of the tail with 10^6^ *Bb* or *Bb-GFP* via subcutaneous needle injection. To determine if there were differences in tissue dissemination between *Bb* and *Bb-*GFP, mice were euthanized on day 14 post-infection, and the spleen, heart, skin near the injection site, joint, bladder, inguinal lymph nodes, and ears were collected and assessed for the presence of spirochetes via BSK-II inoculation (Fig. 2A and B) and qPCR (Fig. 2C through G). All experimentally infected *Bb-* and *Bb-*GFP-challenged mice were culture positive for at least one organ on day 14 post-challenge (Fig. 2A), whereas non-challenged animals were culture negative (data not shown). There was no statistically significant difference in the number of culture-positive organs per mouse between the two groups (Fig. 2A), with averages of 3 to 4 positive samples per mouse for *Bb-* and *Bb*-GFP-challenged mice, respectively. Overall, most bladders, ears, inguinal lymph nodes, and skin samples were culture positive for *Bb-* and *Bb*-GFP-infected mice (Fig. 2B). Joints samples had minimal culture positivity in both conditions, and neither heart nor spleen samples were found to be culture positive in either infection group (Fig. 2B).

In addition to culture positivity, bacterial burden was also assessed by qPCR of isolated DNA from all murine samples (Fig. 2C through G). For assessment of sample integrity, murine beta-actin was used (Fig. S2). Bacterial burden for all samples tested was statistically significantly higher than that of the non-challenged controls. The average spirochete copy number in each organ was variable between *Bb-* and *Bb*-GFP-infected groups, ranging from 900 to 1,900 copies per organ (joint, bladder, and lymph nodes, Fig. 2E through G) and from 190 to 1,500 copies per 5 mg of tissue (ears and skin; Fig. 2C through D). However, differences between groups were not statistically significant between *Bb-* and *Bb*-GFP-infected animals. Overall, these data demonstrate that both *Bb* and *Bb-*GFP strains are capable of infecting mice with similar infection rates and organ dissemination.

### *Bb*-GFP infects ticks in the immersion infection model

Subcutaneous needle inoculations are not representative of the natural route of infection of *B. burgdorferi*; therefore, the *Bb-*GFP strain was evaluated to determine if it could infect the natural tick vector species (*Ixodes*) and be maintained through molting. To determine if the *Bb*-GFP strain was capable of infecting ticks, unfed larval-stage *Ixodes scapularis* ticks were infected by immersion (25) in a *Bb*-GFP suspension. Post-infection, larvae were fed on mice, allowed to molt into the nymph stage, and again fed on mice. DNA was isolated from a subset of ticks, and the presence of *B. burgdorferi* was assessed via qPCR against *23S*. Overall, *B. burgdorferi* PCR-positive samples were identified in both larval and nymphal life stages, with the percentage of *B. burgdorferi*-positive tick samples in larvae increasing post-feeding (61% to 95% positive) (Table 1). After molting into the nymph stage, the percentage of *B. burgdorferi* PCR-positive ticks was 100% (Table 1). To determine whether the *B. burgdorferi* detected in these samples were GFP-positive, we checked for the presence of the GFP plasmid via qPCR. All *B. burgdorferi* PCR-positive unfed larvae, unfed nymphs, and fed nymphs were *gfp* positive, and 78% of *B. burgdorferi* PCR-positive fed larvae were *gfp* positive, indicating maintenance of the plasmid (Table 1). These data suggest that the *Bb-*GFP strain can infect larval ticks in a tick immersion model and that these ticks retain the infection into their nymphal life stage, even after feeding, demonstrating that *Bb*-GFP can be used in tick-immersion-based *B. burgdorferi* studies.

**TABLE 1:** *B. burgdorferi* GFP tick infection

	*Bb* positive (%)	GFP, *Bb* positive (%)
Unfed larva	11/18 (61%)	11/11 (100%)
Fed larva	18/19 (95%)	14/18 (78%)
Unfed nymph post molt	5/5 (100%)	5/5 (100%)
Fed nymph	9/9 (100%)	9/9 (100%)

### Flow cytometry-based culture screening for *B. burgdorferi* is both sensitive and reliable

**Fig 3:**
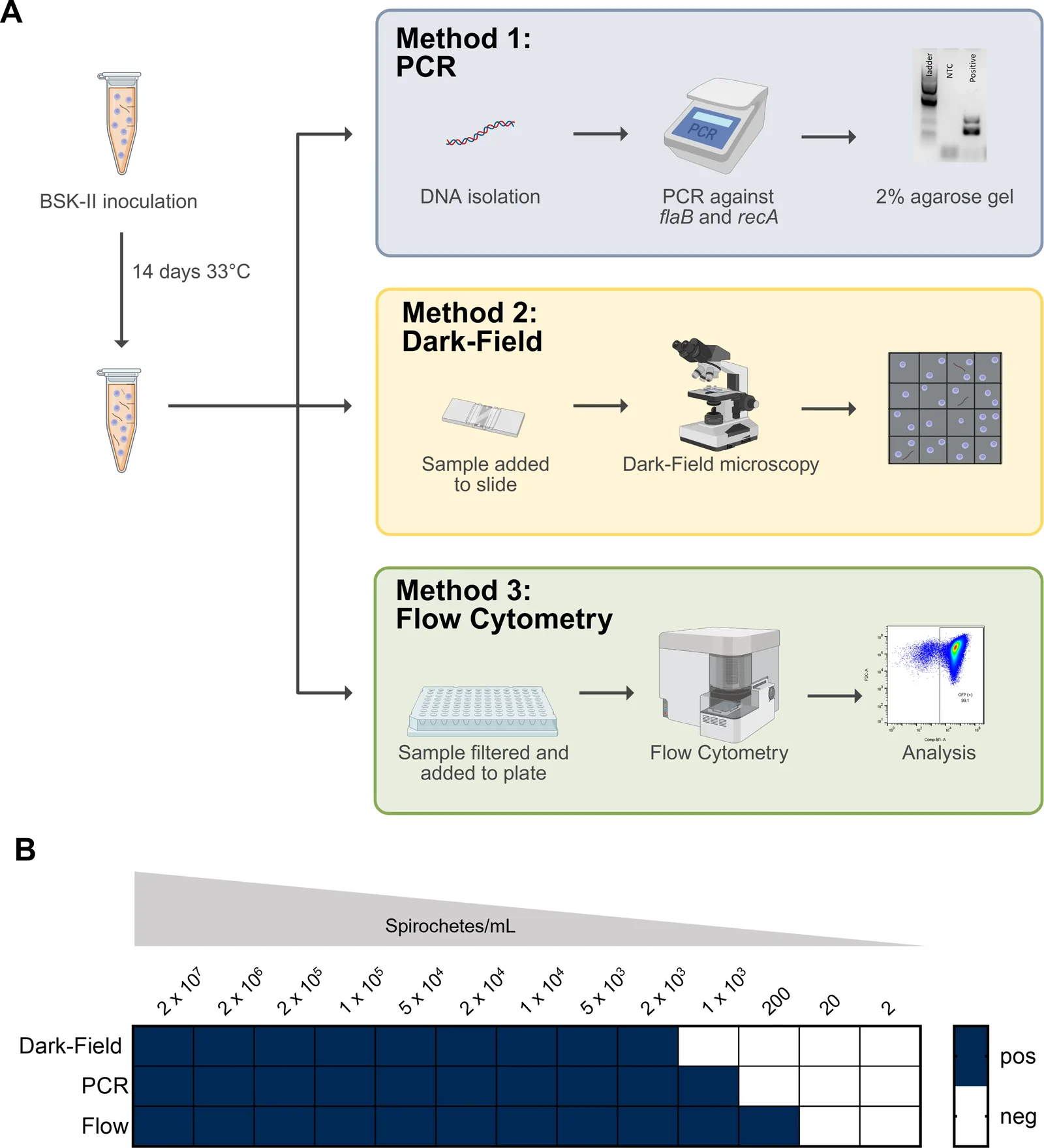
Culture screening methodology and limits of detections. (**A**) Schematic of our BSK-II culture screening process for *Bb-*GFP-infected tissue samples. (**B**) Serial dilutions of *in vitro*-grown *Bb-*GFP (A3-68Δ*bbe02* pBBE22G-flgBpGFP) were performed in BSK-II, and culture positivity was assessed immediately via multiplex PCR against *flaB* and *recA*, dark-field microscopy, and flow cytometry. Colored boxes indicate that 2/3 samples were positive at a given spirochete concentration, whereas white boxes indicate that a sample had 1/3 or lower positive samples at a given spirochete concentration.

Dark-field microscopy is the primary method for determining the presence ofspirochetes in inoculated cultures, but it can be difficult due to tissue debris, low spirochete concentration, time-consuming procedures, and human bias during positivity/negativity assessment (Fig. S3). This study aimed to determine if the fluorescence of the *Bb*-GFP strain could be used to streamline the screening of cultures in a more objective manner (Fig. 3A). To gauge the feasibility of this approach, serial dilutions of *in vitro*-grown *Bb-*GFP cultures were made in BSK-II, and sample positivity was assessed via multiplex PCR against two conserved *B. burgdorferi* genes, *flaB* and *recA*, dark-field microscopy, and flow cytometry. All methodologies were able to detect spirochetes down to 2 × 10^3^ spirochetes/mL, and flow cytometry was found to reliably detect spirochetes at the lowest concentration tested of the three methods, 2 × 10^2^ spirochetes/mL (Fig. 3B).

**Fig 4:**
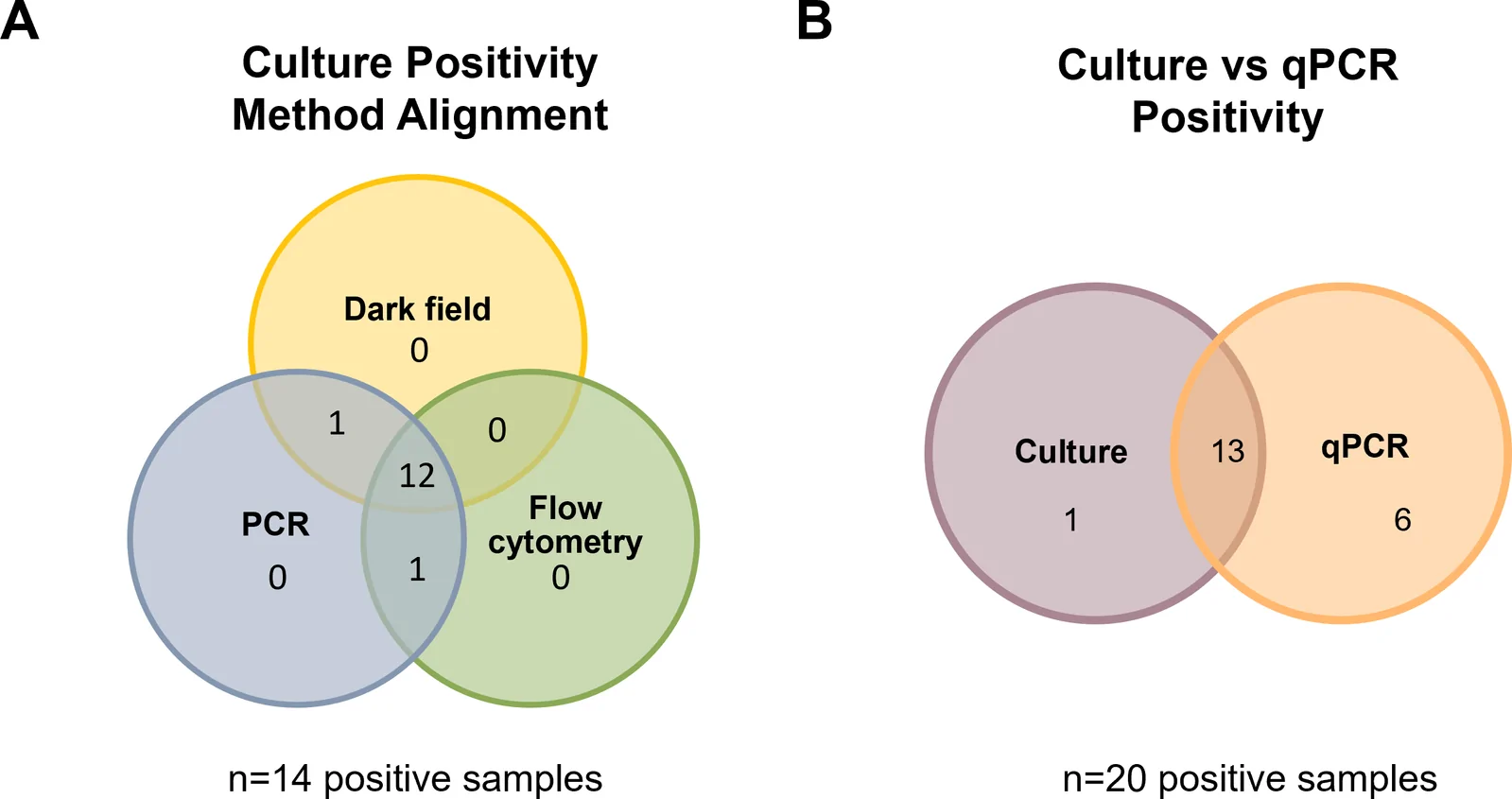
Sample positivity method alignments. Skin, ears, joints, heart, spleen, and bladder were screened for culture positivity from *Bb*-GFP-infected mice euthanized 14-days post-challenge (*n* = 5). (**A**) Venn diagram depicting the concordance of culture positivity determined by either dark-field microscopy, flow cytometry, or PCR. (**B**) Venn diagram depicting culture vs. qPCR sample positivity agreement.

To determine if dark-field microscopy, PCR, or flow cytometry methodologies provided similar results in the context of the *Bb*-GFP murine infection model, culture positivity was assessed by each of the three methods. Mice were challenged with the *Bb*-GFP strain and euthanized 14 days later. Organs were inoculated into BSK-II medium and evaluated for the presence of spirochetes 14 days after inoculation, as depicted in Fig. 3A. We found that flow cytometry was able to detect GFP-positive spirochetes in cultures of infected animals, and in 92% of the samples, these results aligned with positivity detected using either PCR or dark-field microscopy (Fig. 4A). Additionally, we assessed the level of correlation between samples in which one part was inoculated into BSK-II culture and the other portion saved for qPCR on the day of euthanasia (Fig. 4B). A sample was scored as culture positive if it was positive by at least two of the three tested methods, as shown in Fig. 4A. We found that 65% of the samples were consistently positive by both methods (Fig. 4B). Potential discrepancies may lie in differences in the sensitivity of the methodologies as well as in the bacterial viability of the sample, as qPCR does not distinguish between dead and live bacteria. Together, these data suggest that detection of GFP positivity via flow cytometry is a useful tool for streamlining culture screening efforts.

## DISCUSSION

The increase in reported cases of Lyme disease (4), the absence of approved human vaccines, together with the 10%-20% of patients who experience (26) PTLD symptoms, highlight the importance of research with *B. burgdorferi* in preclinical models to develop new interventions. Current preclinical models to study this pathogen are limited by their dependence on time-consuming, labor-intensive, and subjective methods. These limitations are relevant in the context of drug and vaccine development studies, which often utilize large numbers of mice. In this study, we demonstrated that the use of a fluorescent *B. burgdorferi* strain in murine challenge models can help overcome some of the current limitations of traditional studies.

We developed a fluorescent *B. burgdorferi* strain, *Bb*-GFP, and confirmed that *Bb*-GFP expressed GFP *in vitro*, as detected by both flow cytometry and fluorescence microscopy, and that inclusion of the GFP plasmid did not impact bacterial growth compared to its *Bb* parental control strain. When administered to mice using a subcutaneous needle inoculation model of murine infection, both *Bb* and *Bb*-GFP led to 100% infection rates. Bacteria were typically detected in the lymph nodes, bladder, ears, and joints. This is similar to previous work, in which bacteria from the B31 A3-68 strain were detected in bladder, ears, and joints and, in addition, in the heart (27). Differences could be due, in part, to the method of infection (28), the plasmid contents of the strains (Fig. S5), or the dose. In this work, we used a high dose for needle inoculation (10^6^ spirochetes). Given that a broad range of infectious doses are often used in the field (10^2^ to 10^7^) and that the route of delivery can vary (intradermal, subcutaneous, and via tick bite[28–34]), future studies should consider validating the use of GFP-labeled *B. burgdorferi* in the models used in each laboratory. In this study, we demonstrated that the *Bb*-GFP strain could infect larval ticks and that the ticks retained the GFP plasmid through feeding and molting into their nymphal stage. Future work should be conducted to determine whether the bacteria maintain detectable *gfp* expression through the adult stage. A caveat of this study is that ticks were infected using an immersion model of infection. It will be important for future studies to determine whether ticks can acquire the *Bb*-GFP strain through feeding on infected mice and whether infected ticks can then transmit *Bb*-GFP to naïve mice. These future studies will be critical for determining the relevance of this specific strain in models that better mimic the natural zoonotic cycle of this pathogen. Additionally, studies evaluating the potential for detection of GFP fluorescence in *Bb-*GFP should also be performed to determine whether GFP labeling can be used for stain-free detection of this strain. One of the advantages of the design used for the plasmid that encodes GFP in *Bb-*GFP is that the plasmid contains *pncA*. This gene is important for infection in mice (24) and allows for maintenance of the plasmid during murine infections. While transformation with plasmids containing the *pncA* gene are typically used for *pncA* complementation as an *in vivo* genetic mechanism of selection, expression of *pncA* on pBBE22G-*flgBpGFP* facilitates plasmid stability and maintenance in the case of loss of lp25 (24). As demonstrated in this paper, the cultivability of GFP-positive spirochetes from organs in our murine challenge model suggests that the plasmid is maintained during the 14-day period of infection. However, *Bb*-GFP still has the endogenous copy of this gene on plasmid lp25 and may therefore not be a true *in vivo* selection marker for the GFP-encoding plasmid. Knocking out *pncA* on the endogenous lp25 plasmid or adding GFP directly onto the lp25 plasmid may be beneficial to ensure maintenance of the plasmid during murine infection. Alternatively, having a copy of *gfp* in the *B. burgdorferi’s* chromosome instead of in a plasmid could overcome any potential issue regarding plasmid stability.

In this study, we demonstrated that GFP -positive spirochetes were able to be recovered from mice infected with *Bb*-GFP and identified using the flow-based screening methodology outlined in this paper. One of the potential disadvantages of using flow cytometry to detect GFP-expressing bacteria in tissue samples is that tissue or debris may exhibit autofluorescence, which can impact the gating strategy. We tested a wide variety of tissues and confirmed that *Bb*-GFP is distinguishable from tissue debris using our methodology (Fig. S4). Although we confirmed the stability of both the GFP plasmid and *gfp* expression for up to 31 days *in vitro*, this screening method relies on sufficient bacteria maintaining the plasmid in the mouse to be detected by flow cytometry, which may vary from organ to organ. By additionally screening *Bb*-GFP-infected tissue cultures with PCR and dark-field microscopy, we were able to validate our findings and demonstrate that flow cytometry provides a fast and reliable way to screen tissue cultures from animal experiments.

Interestingly, we observed that flow cytometry was more sensitive and could detect *B. burgdorferi* in solutions that were half a log to a log less concentrated than those detected by PCR and dark-field microscopy, respectively (Fig. 3B). Differences in detection limits may reflect differences in the molecular biology of the technique, what is being detected (DNA vs. protein vs. whole spirochete), and the sample volume used for each assay. Flow cytometry allowed for greater sample volume to be assessed in comparison with PCR or dark-field microscopy, resulting in the detection of spirochetes in samples with lower bacterial concentrations. Multiple screenings of low-concentration samples with dark-field microscopy may help increase the chances of detecting spirochetes with this method, albeit at the expense of user time. Increasing the volume and/or concentration of the DNA sample used for PCR may help lower the limit of detection. However, this should be done by increasing the total volume of the PCR reaction, which can be costly, as adding high concentrations of DNA to PCR reactions can inhibit DNA amplification.

Altogether, these data highlight that flow cytometry is a reliable method for assessing culture positivity in murine models of *B. burgdorferi* infection. An advantage of the screening methodology described in this paper is that it was validated using plate-based flow cytometers, enabling users to run one 96-well plate at a time, hands-free. This high-throughput screening capability is especially beneficial in the context of large-scale mouse experiments, in which multiple organs are collected for analysis. The method described here relies on the availability of a fluorescent *B. burgdorferi* strain, such as the one described in this study. However, this culture screening method could be adapted to other fluorescent *B. burgdorferi* strains or additionally used with an anti-*B*. *burgdorferi* antibody.

In summary, this study outlines a workflow to streamline culture screening in preclinical murine models of *B. burgdorferi* infection, using the study-validated A3-68Δ*bbe0*2 pBBE22G-*flgBpGFP* B31 *B. burgdorferi* strain. The methods outlined in this study provide a solution to overcome some of the limitations of current techniques used to study *B. burgdorferi* infection. This workflow, as shown in our previous work, allows for a fast and reliable determination of therapeutic efficacy (35) and demonstrates the advantage of using GFP labeling in the context of murine challenge models of *B. burgdorferi*.

## MATERIALS AND METHODS

### Bacterial strains and growth conditions

Infectious clone A3-68*∆bbe02* (*Bb*), a derivative strain from *B. burgdorferi sensu stricto* B31-A3, was used to generate the GFP strain in this study (23). For the construction of the pBBE22G-*flgBpGFP* vector, the *flgBp-GFP* ORF was PCR amplified from a previously constructed plasmid pFBgfp/T7t (36) using flgBp*-*GFP primer pair (Table S1). The amplicon was then cloned into the pBBE22G vector (24, 37, 38) at PstI and XbaI sites, generating pBBE22G-*flgBpGFP* (NCBI GenBank submission #2989308). Of note, flgBp stands for the *flgB* promoter of *B. burgdorferi* (39). The vector was electroporated into the competent cells of A3-68*∆bbe02* under the selection of streptomycin and gentamicin. Expression of GFP was confirmed by immunoblotting with a GFP antibody and by fluorescence microscopy. GFP-positive bacteria were used to infect naïve 8-week-old BALB/c mice (Jackson Laboratory, Bar Harbor, MN). Post-euthanasia GFP-positive bacteria were isolated from mouse tissue to ensure full infectivity. This mouse-isolated bacteria, named A3-68Δ*bbe02* pBBE22G-flgBpGFP, are herein referred to as either *B. burgdorferi* GFP or *Bb*-GFP in this study. *Bb-*GFP and its parental strain *Bb* were grown as described in Gutierrez et al.(35). Briefly, spirochetes were grown in complete liquid BSK-II medium supplemented with 6% (*v/v*) heat-inactivated rabbit serum (Novus Biological Cat. #S15110) at 33°C and 5% CO_2_. *Bb* cultures were supplemented with streptomycin (50 µg/mL) (Gibco Cat. #11,860-038), and *Bb*-GFP cultures were supplemented with streptomycin (50 µg/mL) and gentamicin (40 µg/mL) (Sigma-Aldrich Cat. #G1264) unless otherwise indicated. Bacteria were enumerated using dark-field microscopy on disposable Neubauer counting chamber slides (Bulldog Bio Cat. #DHC-N01).

### Microscopy

At days 4 and 31 post-inoculation, spirochetes were incubated with 2 µL of DAPI stain (Sigma-Aldrich Cat. #D8417) for 10 min at room temperature, pelleted by centrifugation (6,000 *× g* for 10 min, Sorvall Legend Micro 21 centrifuge), and resuspended in phosphate buffered saline (PBS). Spirochetes were imaged with either a MIF Nikon A1R/SIM confocal microscope or an EVOS FL Imaging System. Images were analyzed in ImageJ 1.48v Fiji.

### qPCR for bacterial DNA quantification

qPCR was performed as described in Gutierrez et al. (35). Briefly, bacteria were pelleted as described above and stored at −20°C. At the time of DNA isolation, samples were incubated with solid tissue buffer (Zymo Cat. #D4068-2-10), nuclease-free water (Thermo Fisher Cat. #AM9937), and proteinase K (Zymo Cat. #D3001-2-125) for 1 h at 55°C. Following proteinase K digestion, DNA was isolated using a Quick-DNA 96 Kit (Zymo Cat. #D3012) following the manufacturer’s instructions, and DNA was eluted in 30 µL of nuclease-free water. Quantitative real-time PCR was performed on isolated DNA using an Applied Biosystems StepOne Plus instrument. iTaq Universal SYBR Green (BioRad Cat. #1725121) enzyme was used, and DNA was amplified using specific primers targeting *gfp* or *B. burgdorferi 23S* (Table S1) with the following cycling conditions: 95°C for 5 min; 45 cycles of denaturation at 95°C for 5 s, with annealing/extension at 60°C for 30 s. Melt curves were additionally run on each sample. For each sample, *23S* or *gfp* copy number was calculated by extrapolation from the standard curve for each primer used.

### Plasmid profiling of strains

To determine the plasmid profile of both strains, PCR was performed on DNA isolated from *Bb* or *Bb*-GFP as described above. Three or 10 ng of DNA was amplified using primers specific to each plasmid (Table S1) and GoTaq G2 Green master mix (Promega Cat. #M7823). The cycling conditions were as follows: initial denaturation at 95°C for 2 min; 35 cycles of denaturation at 94°C for 30 s, annealing at 53–60°C for 30 s, and extension at 72°C for 40 s; followed with a final extension at 72°C or 75°C for 5 min. Specific details on annealing temperatures, DNA used for the PCR reaction, and primer sequences used can be found in the supplementary materials (Fig. S5 and Table S1). PCR products were run on a 2% agarose gel and imaged using a Bio-Rad ChemiDoc Touch imaging system (Fig. S5).

Prior to challenge, *B. burgdorferi* strains were checked for the presence of the following virulence plasmids: lp28-1, lp36, and lp25 (40). Briefly, 100 µL of culture was pelleted at 6,000 × *g* and resuspended in 20 µL of nuclease-free water (Thermo Fisher Cat. #AM9937). This suspension was boiled at 95°C for 10 min. To confirm the presence of each virulence plasmid, PCR was performed on the lysate using primers specific to lp28-1, lp36, or lp25 plasmid (Table S1) and GoTaq G2 Green master mix. The following cycling conditions were used: initial denaturation at 94°C for 2 min; 35 cycles of denaturation at 94°C for 30 s, annealing at 55°C for 30 s, and extension at 72°C for 60 s; followed by a final extension at 72°C for 5 min. PCR products were run and imaged as described above. Cultures were only used for infection if all virulence plasmids were present.

### Murine challenge studies

Three days post-inoculation, cultures of either *Bb* or *Bb*-GFP were pelleted as described above and resuspended in endotoxin-free PBS (Millipore Sigma, Cat. #TMS012A). Ten-week-old female C3H/HeNCrl mice (Charles River Laboratories) were injected with 50 µL (10^6^ spirochetes total) of either *Bb* or *Bb-*GFP or 50 µL PBS subcutaneously at the base of the tail. On day 14 post-infection, mice were euthanized with an intraperitoneal injection of 390 mg of pentobarbital/kg (Patterson Veterinary, Cat. #07-805-9296) in sterile 0.9% w/v NaCl (Baxter, Cat. #2F7124). Blood was collected via cardiac puncture. The right ear, skin surrounding the injection site, bladder, heart, spleen, inguinal lymph nodes, and right joint were aseptically removed. Soft tissue was homogenized using pestles. Approximately half of each organ or 40 µL of organ homogenate was placed in complete liquid BSK-II medium, and the remaining portion was stored at −20°C. Organ cultures were incubated for 14 days at 33°C with 5% CO_2_.

### Assessment of *Borrelia*-positive cultures: dark-field microscopy

Ten microliters of each sample were loaded onto a disposable Neubauer counting chamber slide (Bulldog Bio Cat. #DHC-N01). Nine quadrants of each counting chamber were examined, and a sample was scored as positive if at least one spirochete was found. Contaminated samples were scored as inconclusive and therefore negative for this method.

### Assessment of *Borrelia*-positive cultures: *PCR*

To obtain DNA-containing suspensions, 100 µL of each culture were centrifuged, then resuspended and boiled in 20 µL of water as described earlier. Multiplex PCR with primers for *flaB* and *recA* (Table S1) were performed using GoTaq G2 Green master mix (Promega Cat. #M7823), as described in Gutierrez et al. (35). Thermal cycling was conducted as follows: initial denaturation at 94°C for 2 min; 35 cycles of denaturation at 94°C for 30 s, annealing at 60°C for 30 s, and extension at 72°C for 20 s; followed by a final extension at 72°C for 5 min. PCR products were run on a 2% agarose gel and imaged using a Bio-Rad ChemiDoc Touch imaging system.

### Assessment of *Borrelia*-positive cultures: flow cytometry

Cultures were strained through 70-µm filters to remove debris and diluted 1:10 in PBS. Samples were either run on a BD LSRFortessa or a Cytek Aurora to detect *Bb*-GFP. Non-fluorescent *B. burgdorferi* and tissue from non-infected mice were included as negative controls. Data were analyzed using FlowJo v10.6.1 software, and samples were considered *Bb*-GFP-positive if they contained GFP -positive events greater than or equal to three times those of the negative controls. Ninety-six-well round-bottom plates (Fisher Scientific Cat. #08-772-5) were used for assays run on the Cytek Aurora.

### qPCR on murine tissue samples

First, the joint and skin were homogenized with a pestle in liquid nitrogen to break up the tissue before digestion. Next, half of the joint, 40 µL of bladder and lymph node homogenate, and 5 mg of heart, spleen, ear, or skin were digested with solid tissue buffer (Zymo Cat#D4068-2-10), nuclease-free water (Thermo Fisher Cat. #AM9937), and proteinase K (Zymo Cat# D3001-2-125), for 1–3 h at 55°C. Lastly, DNA for qPCR was isolated and purified as described earlier. qPCR was run as described above, in triplicate, using primers specific for *23S* and *musB* (Table S1). Copy number in each sample was extrapolated from previously generated standard curves.

### Tick immersion infection model

Larval *Ixodes scapularis (Ixs*) ticks were purchased from Oklahoma State and infected following Policastro and Schwan et al. (25) with minimal modifications. *Bb*-GFP was grown to 10^8^ spirochetes/mL, pelleted at 8,000 *× g* for 15 min, and resuspended in 2 mL of BSK-II. Approximately 100 larval ticks were added to this suspension and incubated for 45 min with orbital shaking. After incubation, BSK-II was carefully removed and replaced with 2 mL of water and then shaken for 3 min. Next, this mixture was centrifuged at 200 *× g* for 3 min, and water was removed. Ticks were rinsed with water followed by ethanol and were saved for qPCR, used directly for feeding, or returned to the insectary incubator.

### Tick feeding

For larval feeding, mice were anesthetized with isoflurane. Infected larvae were placed on mice, and mice were monitored daily for signs of distress. Fed larval ticks were collected, and a portion were saved for qPCR, while the remainder were placed back into the insectary incubator. For nymph feedings, ticks were placed on ice to slow them down, and mice were anesthetized with isoflurane. Mice's backs were shaved, and a capsule was attached to their back with a 3:1 rosin (Thermo Fisher, Cat #036,734.A1)-to-beeswax (Millipore Sigma, Cat#243,248) mixture (41). *Bb-*GFP-infected ticks were added to the capsules (five per mouse). Mice were monitored daily for tick detachment, and once a tick was detached, it was removed and saved for downstream analysis in the tick incubator. qPCR of tick samples

Ticks from larval or nymphal stages pre- and post-feeding were frozen with liquid nitrogen and homogenized with a mortar and pestle. Homogenate was resuspended in 180 µL of digestion solution with 20 µL of proteinase K and incubated at 56°C for 1 h (or until complete lysis). Following incubation, the GeneJet Genomic DNA Gram-negative purification kit protocol (Thermo Fisher, Cat. #K0721) was followed, and DNA was eluted in water. For qPCR amplification, PowerTrack SYBR Green Master Mix (Thermo Fisher, Cat. #A46109) was used with primers specific to *Ixodes scapularis beta-actin, gfp,* and *B. burgdorferi 23S* (Table S1). Samples were run on a Bio-Rad CFX machine using the Maestro program with the cycling conditions described above. Copy number was determined using previously established standard curves for each primer tested.

### Statistical analysis

Statistical analysis was performed using GraphPad Prism version 9 (GraphPad). Comparison between two groups was performed by the Mann-Whitney test. Comparisons among three or more groups were analyzed using the Kruskal-Wallis test with Dunnett’s post hoc test for non-parametric data. Comparison with categorical data were performed with Fisher’s exact test.

## Supplementary Material

Reviewer comments

## SUPPLEMENTAL MATERIAL

Supplemental figures and table spectrum.02290-25-s0001.pdf Figures S1 to S5 and Table S1.

OPEN PEER REVIEW reviewer-comments.pdf An accounting of the reviewer comments and feedback.

ASM does not own the copyrights to Supplemental Material that may be linked to, or accessed through, an article. The authors have granted ASM a non-exclusive, world-wide license to publish the Supplemental Material files. Please contact the corresponding author directly for reuse.
