# Supplementary material for: Development of a fluorescent reporter strain to facilitate studies of Borrelia burgdorferi pathogenesis
Source: Microbiol Spectr. 2026 Jun 1;14(7):e02290-25. doi: 10.1128/spectrum.02290-25 (PMC13340187; doi:10.1128/spectrum.02290-25)
Supplement: Supplemental figures and table — Figures S1 to S5 and Table S1. [file spectrum.02290-25-s0001.pdf]

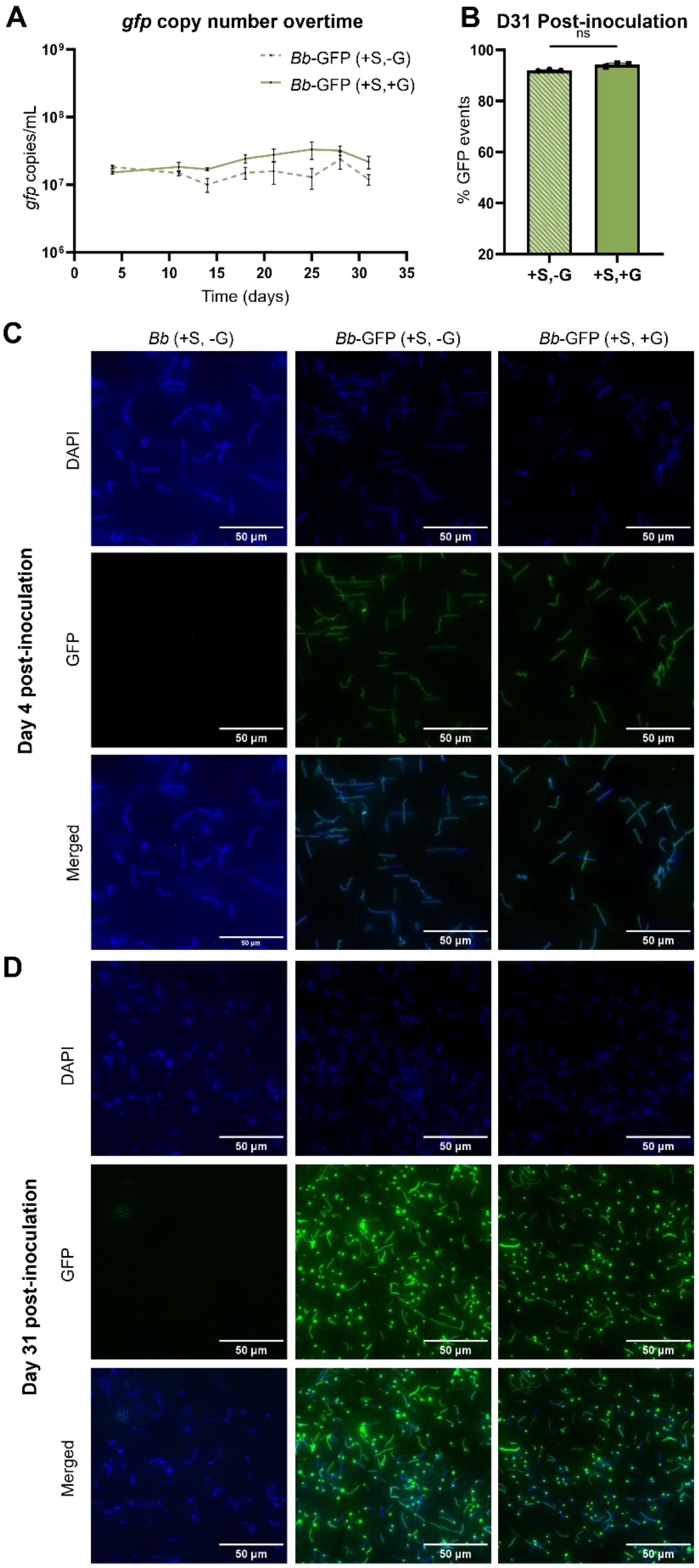

Figure S1. *In vitro* stability of GFP in the presence or absence of selection antibiotic gentamicin. *Bb*-GFP (A3-68Δ*bbe02* pBBE22G-flgBpGFP) inoculated in BSK-II supplemented with either streptomycin 50 µg/mL (+S, -G) or streptomycin 50 µg/mL and gentamicin 40 µg/mL (+S,+G) were grown for 31 days. (A) *gfp* copy number over time was determined via qPCR (n=2-3). Data is represented as means ± SEM. (B) Percent GFP positive bacterial events day 31 post-inoculation was determined via flow cytometry (n=3). Data is represented as means ± SD. Mann-Whitney tests were performed for statistical analyses above. Ns = not significant *P*>0.05. DAPI-stained *B. burgdorferi* were imaged via EVOS FL Imaging System Day 4 (C) and day 31 post-inoculation.

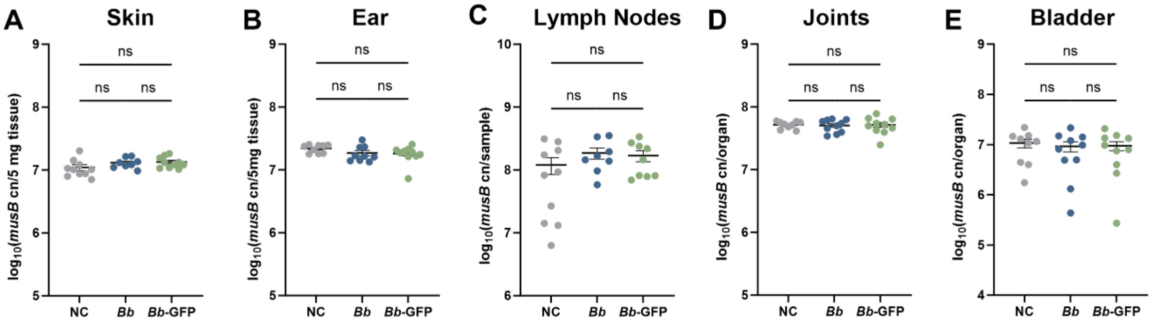

Figure S2. *musB* copies per tissue determined by qPCR for skin (A), ear (B), lymph nodes (C), joints (D), and bladder (E) 14 days post challenge. Data is represented as mean ± SEM. Kruskal-Wallis tests were performed. Ns = not significant, $P>0.05$.

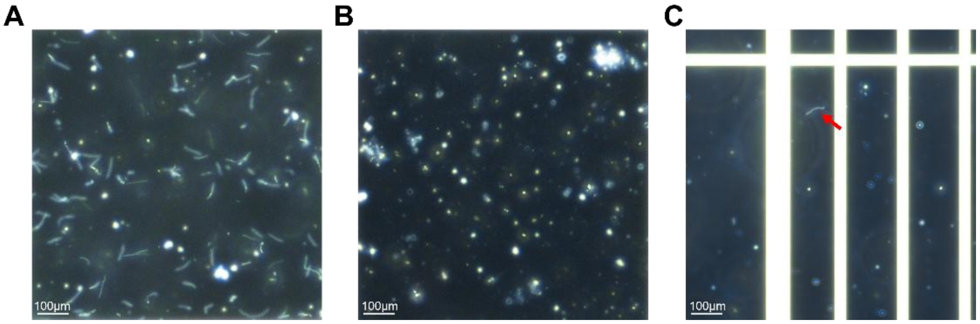

Figure S3. Representative images of culture screening via dark-field microscopy. Images of ¼ diluted samples, imaged with a Nikon ECLIPSE Ci microscope and DS-L4 camera. (A) Represents a culture in which positivity is easily assessed. (B) Represents a culture with an abundance of debris. (C) Represents a culture with minimal spirochetes. Red arrow points to a spirochete in the sample.

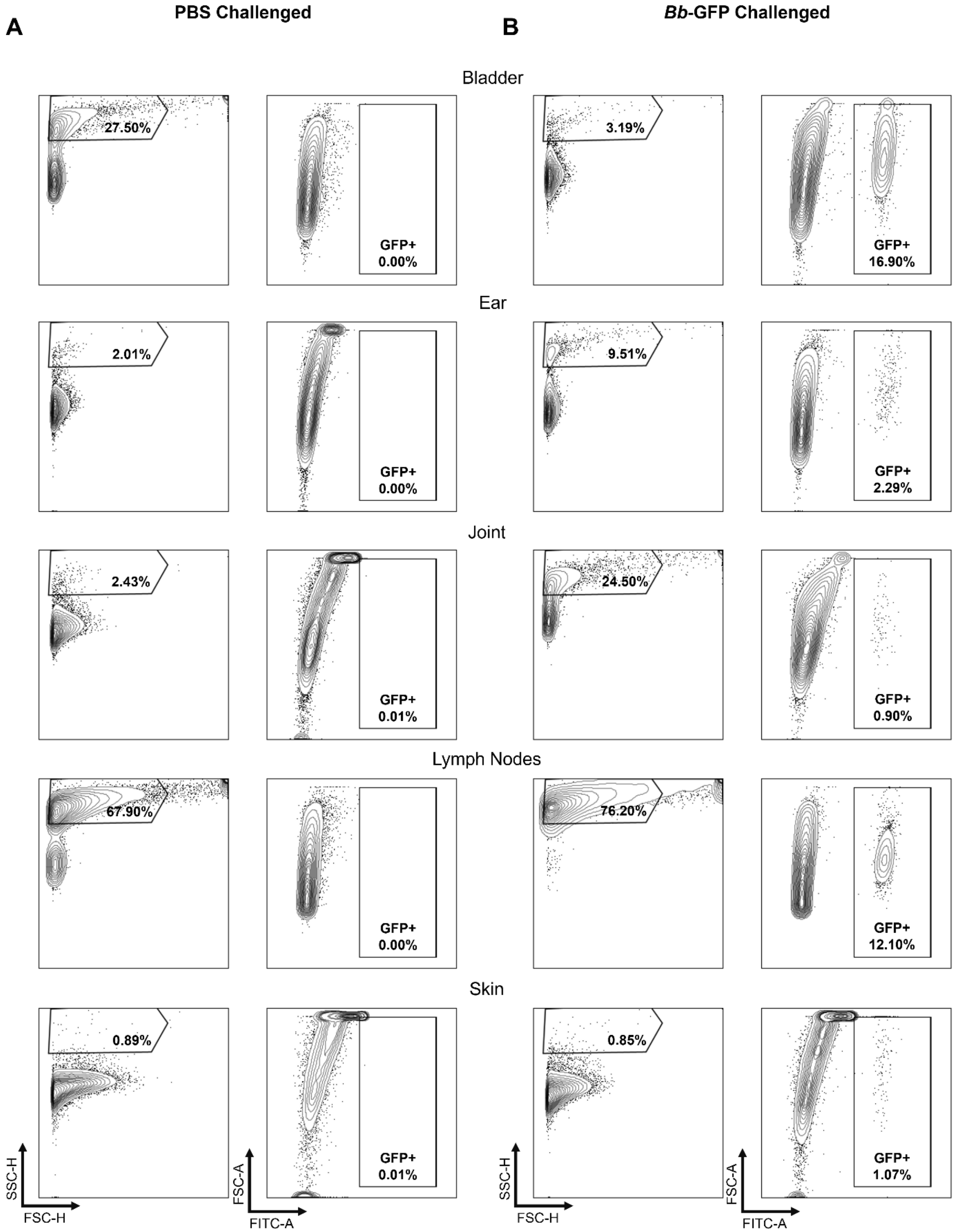

Figure S4. Representative flow cytometry plots showing the gating strategy for each organ of PBS infected (A) or *Bb*-GFP infected (B) mice. Samples were gated based on forward and side scatter (SSC-H vs. FSC-H), then on GFP positivity (FSC-A vs. FITC-A).

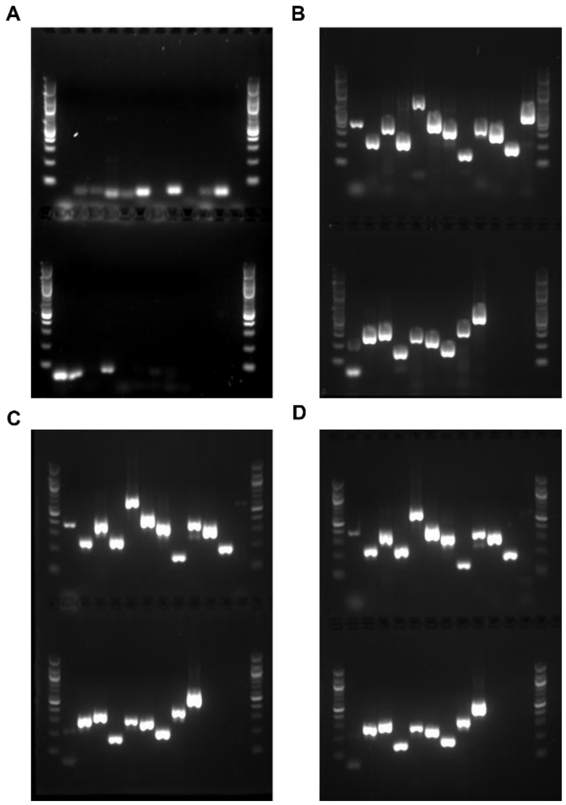

E

Lane	Plasmid	Annealing Temp (°C)	Size (bp)
		Top	
**2**	**lp5**	**53**	**366**
3	lp17	60	250
4	lp21	60	400
5	lp25	55	260
6	lp28-1	55	670
7	lp28-2	60	453
8	lp28-3	60	375
9	lp28-4	60	150
10	lp36	55	381
11	lp38	60	350
12	lp54	60	200
13	lp56	57	596
		Bottom	
**2**	**cp9**	**53**	**226**
3	cp26	60	325
4	cp32-1	60	325
**5**	**cp32-3**	**55**	**170**
6	cp32-4	60	300
7	cp32-6	60	276
8	cp32-7	60	200
9	cp32-8	60	375
**10**	**cp32-9**	**55**	**671**

Figure S5. Plasmid profiles of strains used in this study. PCR negative and positive controls are depicted in (A) and (B), respectively. Plasmid profiles of (C) *Bb* (A3-68Δ*bbe02*) and (D) *Bb*-GFP (A3-68Δ*bbe02* pBBE22G-*flgBpGFP*) strains. PCR reaction annealing temperatures, product lane assignment and expected PCR product sizes are listed in (E). Rows bolded were ran with 10 ng of DNA per reaction, all other reactions were run with 3 ng of DNA per reaction.

Table S1. Primers

Gene/orf amplified		Sequence (5'-->3')	Reference
FlgBp-GFP	Fwd	CTGCAGTACCCGAGCTTCAAGGAAG	Li et al. 2010
	Rev	TCTAGATTATTTGTATAGTTCATCCATGCC	
Bb *recA*	Fwd	GCTGAGGTGCAAAAAGAAGG	Gutierrez et al. 2024
	Rev	GCCTTGCTTGAAGACCAATC	
Bb *flaB*	Fwd	TTTCAGGGTCTCAAGCGTCT	Gutierrez et al. 2024
	Rev	GCTCCTTCCTGTTGAACACC	
Bb *23S*	Fwd	CGAGTCTTAAAAGGGCGATTTAGT	Courtney et al. 2003
	Rev	GCTTCAGCCTGGCCATAAATA	
Mus musculus	Fwd	CGCTCGGTCAGGATCTTCAT	Prantner et al. 2009
β-actin	Rev	TGCCCATCTACGAGGGCTAT	Gutierrez et al. 2024
GFP	Fwd	CTTCGGGCATGGCACTCTT	This study
	Rev	CACTTGTCACTACTTTCGCGT	
Ixodes scapularis	Fwd	GGTATCGTGCTCGACTC	This study
β-actin	Rev	ATCAGGTAGTCGCTCAGG	
Bb lp5	Fwd	ATGAATGGAATAATTAACGATACAC	Elias et al 2002
	Rev	AATATTAGGATGAAGATTATAAATT	
Bb lp17	Fwd	TGTTGGGAAAACTACCTTAAGCGG	Bunikis et al 2011

	Rev	TTGCACATCTCTTCTAAAAGTTCCACTC	
Bb lp21	Fwd	ATGATCATATTTTTATTATCCCCGCAC	Bunikis et al 2011
	Rev	TGAATCTTGCCCTTGACATAACTACC	
Bb lp25	Fwd	AGAATTATGTCGGTGGCGTTGT	Iyer et al. 2003
	Rev	ATTAAAGCCGCCTTTTCCTTGGT	
Bb lp28-1	Fwd	AGTAGTACGACGGGGAAACCA	Iyer et al. 2003
	Rev	ACTTTGCGAACTGCAGAC	
Bb lp28-2	Fwd	CCCTCATCAAGTTTTTCCATGTGTTTTT	Iyer et al. 2003
	Rev	AGGTGGCCTTTCCGAGCTTGTACCTTAC	
Bb lp28-3	Fwd	CTAAGACAGGCCCCGTATGAGG	Bunikis et al 2011
	Rev	GCTAATTGTCGCCACAGTGCTATG	
Bb lp28-4	Fwd	ATGTGGGATGCTATAATCGTGCC	Bunikis et al 2011
	Rev	TTGGTGGTGGCGCAGTGC	
Bb lp36	Fwd	TTCTTATCCCTGACTTTCACTTTCACTTTTGAGG	Iyer et al. 2003
	Rev	TCCTTTACTTCTATGTTTTTACTTTCCTTGGT	
Bb lp38	Fwd	CATCGGCTTTGGTTGCTACATTACC	Bunikis et al 2011
	Rev	GGTTGATTGATGGCTTTCTACGC	
Bb lp54	Fwd	ACGATTGGGACAGCGTTTTAGG	Bunikis et al 2011
	Rev	AACTGAAAAAAGAATTGTGGGAAGAGC	
Bb lp56	Fwd	ACTATTAAGACGAGCAATAAAAAGTCCA	Rego et al 2011
	Rev	GACGAAGCAAAGAAGGATTTGGATCACC	
Bb cp9	Fwd	GATGAACTTGCCGGGGATTG	Bunikis et al 2011
	Rev	CGTATCAAAGGAGTCTTTATACCCAGTG	
Bb cp26	Fwd	GAAGTGGAAGATTGTCAGAAAGAGCC	Bunikis et al 2011
	Rev	CTTCCAGAGTCTGTTCCGCCTG	
Bb cp32-1	Fwd	CATTAAGATTGATGCCGTGGAA	Bunikis et al 2011
	Rev	CTGGGCCTAGAATCGCTGC	
Bb cp32-3	Fwd	ATTGAGGAAGAACTTGAAAAGCTAGC	Bunikis et al 2011
	Rev	ATATCCCCTCCTAGCTTTATTGCC	
Bb cp32-4	Fwd	CTAGCGATATGACCAATGAAGTTATAAC	Bunikis et al 2011
	Rev	TTTTGTGCCTGGGCTCAAAC	
Bb cp32-6	Fwd	TAAAGGATAAGATAGAGAAGTCGGATCC	Bunikis et al 2011
	Rev	CAACTTTCCCTTGATTTTTAGCCC	
Bb cp32-7	Fwd	AACAACAAGACTTTCTGCGAACATC	Bunikis et al 2011
	Rev	GAAAGCTTCTGTAAGTTCCCCTTTAAG	
Bb cp32-8	Fwd	TGAATTTATACCCCATCAAGAGTTGAG	Bunikis et al 2011
	Rev	TCCATATTGAATTCATTATTGCCCG	
Bb cp32-9	Fwd	ACAATTGCCGAAGAACTTGC	This study
	Rev	CCCCCTGATAGGTAGGCTCT	

Work Cited

Bunikis, I., Kutschan-Bunikis, S., Bonde, M. & Bergström, S. Multiplex PCR as a tool for validating plasmid content of Borrelia burgdorferi. *Journal of Microbiological Methods* **86**, 243–247 (2011).

Courtney, J. W., Kostelnik, L. M., Zeidner, N. S. & Massung, R. F. Multiplex Real-Time PCR for Detection of Anaplasma phagocytophilum and Borrelia burgdorferi. *J. Clin. Microbiol.* **42**, 3164–3168 (2004).

Elias, A. F. *et al.* Clonal polymorphism of Borrelia burgdorferi strain B31 MI: implications for mutagenesis in an infectious strain background. *Infect. Immun.* **70**, 2139–2150 (2002).

Gutierrez, M. D. L. P. *et al.* Antibody-mediated immunological memory correlates with long-term Lyme veterinary vaccine protection in mice. *Vaccine* S0264410X24007254 (2024) doi:10.1016/j.vaccine.2024.06.051.

Iyer, R. *et al.* Linear and Circular Plasmid Content in *Borrelia burgdorferi* Clinical Isolates. *Infect. Immun.* **71**, 3699–3706 (2003).

Li, C., Xu, H., Zhang, K. & Liang, F. T. Inactivation of a putative flagellar motor switch protein FliG1 prevents *Borrelia burgdorferi* from swimming in highly viscous media and blocks its infectivity. *Mol. Microbiol.* **75**, 1563–1576 (2010).

Prantner, D. & Nagarajan, U. M. Role for the Chlamydial Type III Secretion Apparatus in Host Cytokine Expression. *Infect. Immun.* **77**, 76–84 (2009).

Rego, R. O. M., Bestor, A. & Rosa, P. A. Defining the Plasmid-Borne Restriction-Modification Systems of the Lyme Disease Spirochete *Borrelia burgdorferi*. J. Bacteriol. 193, 1161–1171 (2011).
