# Supplementary material for: Development of a fluorescent reporter strain to facilitate studies of Borrelia burgdorferi pathogenesis
Source: Microbiol Spectr. 2026 Jun 1;14(7):e02290-25. doi: 10.1128/spectrum.02290-25 (PMC13340187; doi:10.1128/spectrum.02290-25)
Supplement: Reviewer comments [file reviewer-comments.pdf]

AMERICAN SOCIETY FOR MICROBIOLOGY | Microbiology Spectrum

Development of a Fluorescent Reporter Strain to Facilitate Studies of *Borrelia burgdorferi* Pathogenesis

Annalisa Huckaby, Maria de la Paz Gutierrez, Ching wooen Sze, Barrett-Anne Briggs, Kelly Weaver, Evita Yang, Carleena Rocuskie-Marker, Spencer Dublin, Joshua Hall, Samuel Ulicny, Gage Pyles, William Witt, Matthew Hudson, Craig Land, F. Damron, Christopher Pritchett, Chunhao Li, Timothy Driscoll, Emel Sen-Kilic, and Mariette Barbier

Corresponding Author(s): Mariette Barbier, West Virginia University

Review Timeline:

Submission Date:	September 11, 2025
Editorial Decision:	October 2, 2025
Revision Received:	December 15, 2025
Editorial Decision:	February 3, 2026
Revision Received:	April 17, 2026
Accepted:	April 22, 2026

Editor: Ryan Rego

Reviewer(s): Disclosure of reviewer identity is with reference to reviewer comments included in decision letter(s). The following individuals involved in review of your submission have agreed to reveal their identity: Philip E Stewart (Reviewer #2)

Transaction Report:

(Note: With the exception of the correction of typographical or spelling errors that could be a source of ambiguity, letters and reports are not edited. The original formatting of letters and referee reports may not be reflected in this compilation.)

DOI: https://doi.org/10.1128/spectrum.02290-25

Re: Spectrum02290-25 (**Development of a Fluorescent Reporter Strain to Facilitate Studies of *Borrelia burgdorferi* Pathogenesis**)

Dear Dr. Mariette Barbier:

Thank you for the privilege of reviewing your work. Below you will find my comments, instructions from the Spectrum editorial office, and the reviewer comments.

As per the reviewers comments it is clear that modifications are necessary currently for the manuscript submitted. I fully agree with reviewer 2, that plasmid profiles of the parental strain and the GFP clone have to be conducted, given the results presented. Genetic manipulation of any Borrelia strain requires the assessment of the entire set of plasmids for the clones obtained post transformation as well as the parental strain.

Please return the manuscript within 60 days; if you cannot complete the modification within this time period, please contact me. If you do not wish to modify the manuscript and prefer to submit it to another journal, notify me immediately so that the manuscript may be formally withdrawn from consideration by Spectrum.

Revision Guidelines
To submit your modified manuscript, log into the submission site at https://spectrum.msubmit.net/cgi-bin/main.plex. Go to Author Tasks and click the appropriate manuscript title to begin. The information you entered when you first submitted the paper will be displayed; update this as necessary. Note the following requirements:

• Upload point-by-point responses to the issues raised by the reviewers in a file named "Response to Reviewers," NOT in your cover letter.
• Upload a compare copy of the manuscript (without figures) as a "Marked-Up Manuscript" file.
• Upload a clean .DOC/.DOCX version of the revised manuscript and remove the previous version.
• Each figure must be uploaded as a separate, editable, high-resolution file (TIFF or EPS preferred), and any multipanel figures must be assembled into one file.
• Any supplemental material intended for posting by ASM should be uploaded with their legends separate from the main manuscript. You can combine all supplemental material into one file (preferred) or split it into a maximum of 10 files with all associated legends included.

For complete guidelines on revision requirements, see our Submission and Review Process webpage. Submission of a paper that does not conform to guidelines may delay acceptance of your manuscript.

Data availability: ASM policy requires that data be available to the public upon online posting of the article, so please verify all links to sequence records, if present, and make sure that each number retrieves the full record of the data. If a new accession number is not linked or a link is broken, provide Spectrum production staff with the correct URL for the record. If the accession numbers for new data are not publicly accessible before the expected online posting of the article, publication may be delayed; please contact production staff (Spectrum@asmusa.org) immediately with the expected release date.

Publication Fees: For information on publication fees and which article types are subject to charges, visit our website. If your manuscript is accepted for publication and any fees apply, you will be contacted separately about payment during the production process; please follow the instructions in that e-mail. Arrangements for payment must be made before your article is published.

ASM Membership: Corresponding authors may join or renew ASM membership to obtain discounts on publication fees. Need to upgrade your membership level? Please contact Customer Service at Service@asmusa.org.

The ASM Journals program strives for constant improvement in our submission and publication process. Please tell us how we can improve your experience by taking this quick Author Survey.

Thank you for submitting your paper to Spectrum.

Sincerely,
Ryan Rego
Editor
Microbiology Spectrum

Reviewer #1 (Comments for the Author):

In this manuscript, the authors develop and validate a fluorescence-based methodology for the detection of Borrelia burgdorferi

in cultures of infected tissues. The manuscript is very well-written, experimentally sound, and validated. I only have a few minor comments for the authors to address.

Line 186 - Typo = "scapularies"
Line 193-194 - Did the authors test for the presence of Bb-GFP in the ticks via fluorescence microscopy? Stain-free detection of bacteria in ticks is another application that Bb-GFP would be suitable for. Without this data, the concluding statement in lines 198-199 is a bit of an overstatement.
Lines 212-226 - Clarify how many mice were used for this experiment. Also, define the organs tested. Lastly, a representation of the individual tissue results might be useful to include. It would be interesting to see if flow cytometry was sensitive enough to improve detection in "difficult" tissues (e.g., heart, spleen, and joint).
Discussion - The beginning of the Discussion (lines 228-266) largely rehashes the limitations they sought to address. I recommend shortening this and moving any critical background information to the Introduction.

Reviewer #2 (Comments for the Author):

This manuscript by Huckaby et al. details the use of a fluorescent strain of Borrelia burgdorferi and its use in infection studies. The authors characterize 3 different methods to detect B. burgdorferi in various mouse and tick tissues (darkfield microscopy, PCR, and flow cytometry) and conclude that flow cytometry is the most sensitive. Overall, the manuscript is logically presented and is largely thorough in the analyses performed. I have some recommendations to improve the clarity of the manuscript to make it easier for researchers to use this methodology. Importantly, comments 4 and 7 are critical to make this a more rigorous manuscript that can be fully interpreted by researchers. Finally, the authors should be more careful in their use of adverbs and adjectives throughout the manuscript. Specific words that are repeatedly used are misleading.
1. B. burdorferi is not a gram-negative bacteria as mentioned in lines 24 and 70, but a diaderm that lacks LPS.
2. Ln 80: "thoroughly investigated" is not accurate, if it were there would be no need for more research.
3. Ln 100: I would include Carrol et al. Microbiology (2003), 149, 1819-1828 in your list of references.
4. Ln 121, Fig 1A and generally throughout the manuscript: description and antibiotic use for pBBE22G is confusing. As referenced, the backbone vector is pBBE22 (Purser et al. Mol. Microbiol. 2003), not pBBE22G, so where does the "G" come from (gentamicin presumably)? pBBE22 is a shuttle vector that is kanamycin-resistant but the manuscript states that gentamicin is used, please clarify. Also, in figure 1A, the plasmid map shown does not appear to be the pBBE22 backbone and the annotations provided are not explained in the fig legend nor are they correct for the backbone shuttle vector of pBSV2, from which pBBE22 was derived. These inconsistencies must be corrected for anyone to understand the methodology or to recreate this construct.
5. Ln 124: "BBE22 gene" uses the protein form of the name rather than the gene, "BBE22" should be lower case and italicized.
6. Ln 165: should be fig 2A, not 1A as written in text.
7. Ln 170-1 and fig 2: It is unusual that joints, heart, and spleen cultures were either negative or only positive in a minority of the samples. The plasmid profiles of both the Bb parental strain and Bb-GFP should be determined and reported in this manuscript. Plasmid loss might help explain the observed tissue tropism. If the plasmid profiles are identical to A3-68, then the authors should devote some space in the discussion to address this strange tropism.
8. Lns 194-5: "For all stages ... except the fed larval stage". "Stage" generally refers to either larvae, nymph or adult, not the fed state. Also, since only 2 stages were examined (larval and nymphal) it does not make grammatical sense to state "all stages ... except".
9. Please address why fluorescent microscopy was not used to confirm the expression of GFP in tick tissues. Typically, the salivary glands and the midgut do not produce such strong autofluorescence that GFP cannot be detected. While the PCR data is convincing, it would enhance the manuscript to show that GFP is being produced.
10. Ln 206: "4 day old" but figure states 14 days. I assume the figure is wrong but ensure that the manuscript is consistent.
11. Ln 238 "Most" conflicts with subsequent lines 254 and 259 which state other studies "typically assessed". Refrain from using generalized modifiers that are inaccurate or misleading.
12. Ln 278: "detectable GFP-expression". Again, you are referring to the gene here, not the protein, so use lower-case, italics for gfp.
13. Ln 280 and subsequent sentences: It is not clear to me anywhere in this paper why the plasmid used in this study contains pncA. The parental strain contains the endogenous copy so why was this gene included in the plasmid construct? Please address in the manuscript.
14. Ln 369: should be Table S1
15. Fig 1A legend: please define the gene annotations or only show and define relevant genes.
16. Lns 608 and 646: "overtime" should be two separate words.
17. Fig 2 Organ positivity distribution: I assume each row is an individual mouse, if so please indicate such in the figure.
18. Fig 2 legend: define NC
19. Table 1: delete "no molt" from fed nymph line to be consistent within the table.
20. Fig 3A: 14 days 33ºC or 4 days?
21. Fig S1: include merged images as DAPI staining is faint.

Response to Reviewers

Dear reviewers,

Thank you for your careful review of the manuscript. Your comments were very useful and we have modified the manuscript accordingly. For quick reference, the sections modified are referenced using line numbers in the version resubmitted with track changes.

Reviewer #1 (Comments for the Author):

In this manuscript, the authors develop and validate a fluorescence-based methodology for the detection of Borrelia burgdorferi in cultures of infected tissues. The manuscript is very well-written, experimentally sound, and validated. I only have a few minor comments for the authors to address.

Line 186 - Typo = "scapularies".

Corrected, **line 203.**

Line 193-194 - Did the authors test for the presence of Bb-GFP in the ticks via fluorescence microscopy? Stain-free detection of bacteria in ticks is another application that Bb-GFP would be suitable for. Without this data, the concluding statement in lines 198-199 is a bit of an overstatement.

We were unable to perform these experiments due to technical limitations. The text has been modified **lines 207-217** to reflect that the conclusions are based on detection via PCR. Additional discussion has been added **lines 299-301** to highlight the potential of this technology for stain-free detection of *B. burgdorferi*.

Lines 212-226 - Clarify how many mice were used for this experiment. Also, define the organs tested. Lastly, a representation of the individual tissue results might be useful to include. It would be interesting to see if flow cytometry was sensitive enough to improve detection in "difficult" tissues (e.g., heart, spleen, and joint).

We modified the Figure 4 figure legend; now it states how many mice were used (n=5) and which organs were tested (skin, ears, joints, heart, spleen, and bladder) (**lines 674-676)**. We did not see many differences between flow cytometry and other methods in the more “difficult” tissues (see table below). However, we did not find that in our hands heart or spleen to be positive by culture in any of the samples tested.

Culture Positivity

	PCR	Dark-field	Flow
Heart	0	0	0
Bladder	5	5	5

Spleen	0	0	0
Ears	2	1	2
Joints	3	3	2
Skin	4	4	4

Discussion - The beginning of the Discussion (lines 228-266) largely rehashes the limitations they sought to address. I recommend shortening this and moving any critical background information to the Introduction.

We modified the discussion as suggested (**lines 255-282**) and the introduction (**lines 87-108**).

Reviewer #2 (Comments for the Author):

This manuscript by Huckaby et al. details the use of a fluorescent strain of Borrelia burgdorferi and its use in infection studies. The authors characterize 3 different methods to detect B. burgdorferi in various mouse and tick tissues (darkfield microscopy, PCR, and flow cytometry) and conclude that flow cytometry is the most sensitive. Overall, the manuscript is logically presented and is largely thorough in the analyses performed. I have some recommendations to improve the clarity of the manuscript to make it easier for researchers to use this methodology. Importantly, comments 4 and 7 are critical to make this a more rigorous manuscript that can be fully interpreted by researchers. Finally, the authors should be more careful in their use of adverbs and adjectives throughout the manuscript. Specific words that are repeatedly used are misleading.

The text has been revised accordingly.

1. B. burdorferi is not a gram-negative bacteria as mentioned in lines 24 and 70, but a diaderm that lacks LPS.

Corrected **(lines 24 and 70).**

2. Ln 80: "thoroughly investigated" is not accurate, if it were there would be no need for more research.

Corrected **(line 86).**

3. Ln 100: I would include Carrol et al. Microbiology (2003), 149, 1819-1828 in your list of references.

Thank you for the suggestion. It has been added to the reference list **(line 119, reference #22**).

4. Ln 121, Fig 1A and generally throughout the manuscript: description and antibiotic use for pBBE22G is confusing. As referenced, the backbone vector is pBBE22 (Purser et al. Mol. Microbiol. 2003), not pBBE22G, so where does the "G" come from (gentamicin presumably)? pBBE22 is a shuttle vector that is kanamycin-resistant but the manuscript states that gentamicin is used, please clarify. Also, in figure 1A, the plasmid map shown does not appear to be the pBBE22 backbone and the annotations provided are not explained in the fig legend nor are they correct for the backbone shuttle vector of pBSV2, from which pBBE22 was derived. These inconsistencies must be corrected for anyone to understand the methodology or to recreate this construct.

Thank you for pointing this out. The map in Fig 1A has been corrected and simplified, and the figure legend has been modified accordingly. The pBBE22G vector was previously constructed by replacing the kanamycin resistance cassette of the pBBE22 vector with a gentamicin resistance cassette. In the text we additionally modified pBBE22 with pBBE22G where stated incorrectly (**lines 30, 31,32, 350, and 643-646**).

5. Ln 124: "BBE22 gene" uses the protein form of the name rather than the gene, "BBE22" should be lower case and italicized.

Corrected (**line 143 and line 145**)

6. Ln 165: should be fig 2A, not 1A as written in text.

Thank you for noticing this mistake. It has been corrected (**line 183**).

7. Ln 170-1 and fig 2: It is unusual that joints, heart, and spleen cultures were either negative or only positive in a minority of the samples. The plasmid profiles of both the Bb parental strain and Bb-GFP should be determined and reported in this manuscript. Plasmid loss might help explain the observed tissue tropism. If the plasmid profiles are identical to A3-68, then the authors should devote some space in the discussion to address this strange tropism.

Differences have been noted in the field in terms of infection kinetics and tissue tropism depending on the model of infection (ticks vs needle) (Sertour et al). For B31-A3, this strain has reported tropism to liver, heart, ear and bladder. While presence of the GFP strain was not determined in the liver, it was shown to be present in the ear and the bladder, but not in the heart. As noted by this reviewer, these differences could be due to the presence of different plasmid content. To rule this out, we looked at plasmid contents for both strains. The plasmid content is consistent with the literature and the data is now included in Supplementary Figure S5 and the text has been modified **(lines 287-291).**

8. Lns 194-5: "For all stages ... except the fed larval stage". "Stage" generally refers to either larvae, nymph or adult, not the fed state. Also, since only 2 stages were examined (larval and nymphal) it does not make grammatical sense to state "all stages ... except".

Corrected (**lines 211-214**)

9. Please address why fluorescent microscopy was not used to confirm the expression of GFP in tick tissues. Typically, the salivary glands and the midgut do not produce such strong autofluorescence that GFP cannot be detected. While the PCR data is convincing, it would enhance the manuscript to show that GFP is being produced.

Thank you for raising this issue. We unfortunately are not technically equipped to do these experiments and were not able to confirm presence of GFP-positive *B. burgdorferi* in ticks using fluorescence microscopy. The text has been modified **lines 209-217** to reflect that the conclusions are based on detection via PCR exclusively. Additional discussion has been added **lines 299-301** to highlight the potential of this technology for stain-free detection of *B. burgdorferi* and the need for further studies to determine this.

10. Ln 206: "4 day old" but figure states 14 days. I assume the figure is wrong but ensure that the manuscript is consistent.

We modified the figure legend and text for clarification (lines 224-226, line 234 and lines 667-670).

11. Ln 238 "Most" conflicts with subsequent lines 254 and 259 which state other studies "typically assessed". Refrain from using generalized modifiers that are inaccurate or misleading.

Thank you for your suggestion. We modified the text accordingly. This section was moved to the introduction, and the sentence was modified to remove "most" (**line 88**).

12. Ln 278: "detectable GFP-expression". Again, you are referring to the gene here, not the protein, so use lower-case, italics for gfp.

Corrected (**line 298**).

13. Ln 280 and subsequent sentences: It is not clear to me anywhere in this paper why the plasmid used in this study contains pncA. The parental strain contains the endogenous copy so why was this gene included in the plasmid construct? Please address in the manuscript.

Constructs that include *pncA* are meant to be used in strains with deletion of this gene to ensure selection through functional complementation. However, even in strains like the one used in this study that have intact *pncA*, presence of this gene on the plasmid can help with plasmid maintenance and stability in case of loss of lp25 (Purser et al., 2003 (Mol. Microbiol.). This is now discussed **lines 304-307**.

14. Ln 369: should be Table S1.

Thank you for noticing this mistake. It has been corrected (**line 394**).

15. Fig 1A legend: please define the gene annotations or only show and define relevant genes.

Thank you for the suggestion. We simplified the plasmid schematic in Figure 1A, and defined gene annotations in the figure legend (**lines 643-646**).

16. Lns 608 and 646: "overtime" should be two separate words.

Corrected throughout the manuscript (**line 160 and 647)**. This was additionally adjusted in Figure 1B header.

17. Fig 2 Organ positivity distribution: I assume each row is an individual mouse, if so please indicate such in the figure.

Corrected in figure legend **(lines 656-658)**.

18. Fig 2 legend: define NC.

Corrected **(lines 659-660)**.

19. Table 1: delete "no molt" from fed nymph line to be consistent within the table. Corrected **(line 644**).

20. Fig 3A: 14 days 33ºC or 4 days?

Thank you for noticing this. For *Bb*-GFP infected mice, 14 days post-challenge portions of each tissue were inoculated into BSK-II. 14-days later culture positivity was assessed through the three methodologies depicted in Figure 3A. For Figure 3B, *Bb*-GFP was incubated at 33ºC for 4 days, then serial dilutions were performed in BSK-II. Following this, each dilution was screened for positivity using the 3 methods depicted in Figure 3A. The text in the figure legend and the results sections has been modified for clarification **(lines 224-226, line 234 and lines 667-670).**

21. Fig S1: include merged images as DAPI staining is faint.

Thank you for the suggestion, Figure S1 was adjusted accordingly.

Re: Spectrum02290-25R1 (**Development of a Fluorescent Reporter Strain to Facilitate Studies of *Borrelia burgdorferi* Pathogenesis**)

Dear Dr. Mariette Barbier:

Thank you for the privilege of reviewing your work. Below you will find my comments, instructions from the Spectrum editorial office, and the reviewer comments.

Although both reviewers are satisfied with answering of their questions , there are a couple of points that still need to be rectified.

Lines 121-122, please change to read 'only in larvae and nymphs' as adult ticks were never looked at, so saying all life stages is not true. Do this if this is mentioned elsewhere in the manuscript.

I would like you to address in the results and discussion section,

1. Why infected mice were not used for looking at acquisition of the GFP expressing Borrelia by allowing unfed larval ticks to feed on them , given that this is the natural route for infecting ticks. An immersion method is usually used if ticks are unable to naturally acquire Borrelia from an infected animal. Looking at figure 2 (B,E, D), it appears that the GFP expressing Borrelia outside of the injection site were not that capable of being cultured from a distant site like the ear. Was this a possible reason?

Also Line 432 mentions adult tick feedings, is this a mistake?

2. What was the result of transmission to mice by the unfed infected nymphs that had fed on them and if not done, the reason why it was not carried out? I am surprised this has not been discussed.

3. The parental strain A3-68Δbbe02 lacks lp56, yet your plasmid profile shows its presence in this strain as well as the GFP expressing strain. Please comment.

Please return the manuscript within 60 days; if you cannot complete the modification within this time period, please contact me. If you do not wish to modify the manuscript and prefer to submit it to another journal, notify me immediately so that the manuscript may be formally withdrawn from consideration by Spectrum.

Revision Guidelines
To submit your modified manuscript, log into the submission site at https://spectrum.msubmit.net/cgi-bin/main.plex. Go to Author Tasks and click the appropriate manuscript title to begin. The information you entered when you first submitted the paper will be displayed; update this as necessary. Note the following requirements:

• Upload point-by-point responses to the issues raised by the reviewers in a file named "Response to Reviewers," NOT in your cover letter.
• Upload a compare copy of the manuscript (without figures) as a "Marked-Up Manuscript" file.
• Upload a clean .DOC/.DOCX version of the revised manuscript and remove the previous version.
• Each figure must be uploaded as a separate, editable, high-resolution file (TIFF or EPS preferred), and any multipanel figures must be assembled into one file.
• Any supplemental material intended for posting by ASM should be uploaded with their legends separate from the main manuscript. You can combine all supplemental material into one file (preferred) or split it into a maximum of 10 files with all associated legends included.

For complete guidelines on revision requirements, see our Submission and Review Process webpage. Submission of a paper that does not conform to guidelines may delay acceptance of your manuscript.

Data availability: ASM policy requires that data be available to the public upon online posting of the article, so please verify all links to sequence records, if present, and make sure that each number retrieves the full record of the data. If a new accession number is not linked or a link is broken, provide Spectrum production staff with the correct URL for the record. If the accession numbers for new data are not publicly accessible before the expected online posting of the article, publication may be delayed; please contact production staff (Spectrum@asmusa.org) immediately with the expected release date.

Publication Fees: For information on publication fees and which article types are subject to charges, visit our website. If your manuscript is accepted for publication and any fees apply, you will be contacted separately about payment during the production process; please follow the instructions in that e-mail. Arrangements for payment must be made before your article is published.

ASM Membership: Corresponding authors may join or renew ASM membership to obtain discounts on publication fees. Need to upgrade your membership level? Please contact Customer Service at Service@asmusa.org.

The ASM Journals program strives for constant improvement in our submission and publication process. Please tell us how we can improve your experience by taking this quick Author Survey.

Thank you for submitting your paper to Spectrum.

Sincerely,
Ryan Rego
Editor
Microbiology Spectrum

Reviewer #1 (Comments for the Author):

The authors adequately addressed my concerns; no further comments or suggestions are needed.

Reviewer #2 (Comments for the Author):

All my comments have been addressed satisfactorily.

Dear Dr. Rego,

Thank you for your revision of the manuscript. Your comments and corrections were very helpful, and we have modified the manuscript accordingly.

1. Lines 121-122, please change to read 'only in larvae and nymphs' as adult ticks were never looked at, so saying all life stages is not true. Do this if this is mentioned elsewhere in the manuscript.

Thank you for pointing out this mistake. The text was edited accordingly (**lines 120-121; 197-198, 438**).

Points to address in the results and discussion sections:

2. Why infected mice were not used for looking at acquisition of the GFP expressing Borrelia by allowing unfed larval ticks to feed on them, given that this is the natural route for infecting ticks. An immersion method is usually used if ticks are unable to naturally acquire Borrelia from an infected animal. Looking at figure 2 (B,E, D), it appears that the GFP expressing Borrelia outside of the injection site were not that capable of being cultured from a distant site like the ear. Was this a possible reason? (Address in results and discussion)

Thank you for your comment, the text (**lines 208; 260-265**) has been modified accordingly to reflect this limitation in the work presented here. Unfortunately, due to technical limitations, we were not able to perform this experiment and do not currently have the resources to do so. The text was amended to include the importance of adding this experiment to future studies.

3. Also Line 432 mentions adult tick feedings, is this a mistake?

Thank you for pointing out this mistake, the text was modified accordingly (**line 438**).

4. What was the result of transmission to mice by the unfed infected nymphs that had fed on them and if not done, the reason why it was not carried out? I am surprised this has not been discussed. (Address in results and discussion)

Thank you for your comment. For this study the unfed infected nymphs were fed on a small number of naïve mice (n=3). Fourteen days after ticks had molted, we assessed mice for the presence of bacteria using culture and fluorescence microscopy. Results of this preliminary experiment were inconclusive. We were unable to culture spirochetes out of the organs of these mice tested. However, with fluorescent microscopy and staining with an anti-*B. burgdorferi*-fluorescently conjugated antibody, we did find some structures that were both anti-*B. burgdorferi* positive and GFP-positive only in infected samples.

As tick-murine infection models are outside of the expertise of both our and our collaborators labs, we chose not to include that data in this paper as we felt that additional studies would need

to be performed to optimize this model and draw conclusions from it. (i.e. more mice tested, collections of organs at different times post-infection, using a strain of *B. burgdorferi* known to be transmitted through tick infection, etc). These experiments were not performed due to funding/personal limitations. This is a limitation of the paper and has been reflected in text additions (**lines 260-265**).

5. The parental strain A3-68Δbbe02 lacks lp56, yet your plasmid profile shows its presence in this strain as well as the GFP expressing strain. Please comment. (Address in results and discussion)

Thank you for your comment. The plasmid profile for the GFP strain was reran and new gels were added to the supplemental figure (**Figure S5**). The text was edited accordingly (**lines 341-343, 363-383**).

Additionally, the plasmid size in **Figure 1A** was corrected.

Re: Spectrum02290-25R2 (**Development of a Fluorescent Reporter Strain to Facilitate Studies of *Borrelia burgdorferi* Pathogenesis**)

Dear Dr. Mariette Barbier:

Thank you for making the suggested edits and providing the answers to my questions.

Your manuscript has been accepted, and I am forwarding it to the ASM production staff for publication. Your paper will first be checked to make sure all elements meet the technical requirements. ASM staff will contact you if anything needs to be revised before copyediting and production can begin. Otherwise, you will be notified when your proofs are ready to be viewed.

Data Availability: ASM policy requires that data be available to the public upon online posting of the article, so please verify all links to sequence records, if present, and make sure that each number retrieves the full record of the data. If a new accession number is not linked or a link is broken, provide production staff with the correct URL for the record. If the accession numbers for new data are not publicly accessible before the expected online posting of the article, publication may be delayed; please contact ASM production staff immediately with the expected release date.

Publication Fees: For information on publication fees and which article types have charges, please visit our website. We have partnered with Copyright Clearance Center (CCC) to collect author charges. If fees apply to your paper, you will receive a message from no-reply@copyright.com with further instructions. For questions related to paying charges through RightsLink, please contact CCC at ASM_Support@copyright.com or toll free at +1-877-622-5543. CCC makes every attempt to respond to all emails within 24 hours.

ASM Membership: Corresponding authors may join or renew ASM membership to obtain discounts on publication fees. Need to upgrade your membership level? Please contact Customer Service at Service@asmusa.org.

Embargo Policy: A press release may be issued as soon as the manuscript is posted on the Spectrum Latest Articles webpage. The corresponding author will receive an email with the subject line "ASM Journals Author Services Notification" when the article is available online.

The ASM Journals program strives for constant improvement in our submission and publication process. Please tell us how we can improve your experience by taking this quick Author Survey.

Thank you for submitting your paper to Spectrum.

Sincerely,
Ryan Rego
Editor
Microbiology Spectrum
